# Social network for guardians of transgender children and adolescents

**DOI:** 10.1590/S2237-96222024v33e2024290.especial.en

**Published:** 2025-01-13

**Authors:** Paula Daniella de Abreu, Ana Beatriz Marques Valença, Gilberto da Cruz Leal, Diogo Henrique Mendes da Silva, Pedro Fredemir Palha, Ednaldo Cavalcante de Araújo, Jaqueline Garcia de Almeida Ballestero, Sandra Aparecida de Almeida, Jordana de Almeida Nogueira, Aline Aparecida Monroe

**Affiliations:** 1 Universidade de São Paulo, Escola de Enfermagem de Ribeirão Preto, SP, Brasil; 2 Universidade Federal de Pernambuco, Departamento de Enfermagem, Recife, PE, Brasil; 3 Universidade Federal da Paraíba, Departamento de Enfermagem em Saúde Coletiva, João Pessoa, PB, Brasil; 4 Universidade Federal da Paraíba, Departamento de Enfermagem Clínica, João Pessoa, PB, Brasil

**Keywords:** Red Social, Personas Transgénero, Identidad de Género, Niño, Adolescente, Social Network, Transgender People, Gender Identity, Child, Adolescent

## Abstract

**Objective:**

To analyze the social network of mothers, fathers or guardians of transgender children or adolescents.

**Methods:**

This was a qualitative study, based on the theoretical framework of social network, with a focus on the primary network. The study was conducted in Brazil through online interviews between August and October 2021. A total of 30 mothers, two fathers and one grandmother of transgender children or adolescents participated in the study. The thematic content analysis was performed using IraMuTeQ software.

**Results:**

The theme “The family as the center of the network and the challenges in achieving transgender autonomy” emerged from the analyses. The family was identified as the first network, bearing the greatest responsibility. Weak and conflicted ties with relatives, friends, classmates and neighbors, highlighting the role of men.

**Conclusion:**

The networks showed limitations in providing support and the need for strengthening. The analysis is an important tool for improving care, structuring policies and developing transgender-specific care pathways.

## Study contributions

**Main results:**

The support networks of these families, especially the immediate family, play a significant role, but also show vulnerabilities, such as conflicting bonds and a lack of adequate support from other community members.

**Implications for services:**

A more structured and specific approach to supporting families of transgender children and adolescents is essential. Health services should enhance family support, provide tailored assistance, and train healthcare professionals to effectively care for this population.

**Perspectives:**

Future research should investigate interventions that minimize conflicts and strengthen bonds, in addition to evaluating the impact of trans-specific policies on the autonomy and well-being of transgender children and adolescents, thereby guiding the development of more inclusive public policies.

## RESUMEN

**Objetivo:**

Analizar la red social de madres, padres o responsables de niños y adolescentes transgénero.

**Métodos:**

Se trata de un estudio cualitativo, basado en el marco de referencia de la red social, con enfoque en la red primaria. El estudio se llevó a cabo en Brasil a través de entrevistas en línea entre agosto y octubre de 2021. Participaron 30 madres, dos padres y una abuela de niños, o adolescentes transgénero. Se realizó un análisis de contenido temático con la ayuda del software IRaMuTeQ.

**Resultados:**

El tema “La familia como centro de la red y los desafíos para alcanzar la autonomía trans” surgió a partir de los análisis. La familia se configuró como la primera red, con una mayor responsabilidad. Se revelaron vínculos frágiles y conflictivos con familiares, amigos, colegas y vecinos, destacando la figura del hombre.

**Conclusión:**

Las redes presentaron limitaciones en el apoyo y la necesidad de fortalecimiento, siendo el análisis una herramienta importante para la asistencia, la estructuración de políticas y la implementación de una línea de atención específica para personas trans.

## INTRODUCTION

The challenges faced by guardians of transgender children or adolescents, or trans, initially involved the process of recognizing transgenderness. This lack of knowledge can lead to experiences of denial and transphobia, enhanced by feelings of doubt, helplessness in the face of challenges, fear, sadness and guilt.^1-3^ These feelings can also be associated with the experience of “ambiguous loss”, which is understood as the psychological and identity loss of a person who is still physically present.^4^

Gender identity can change existing family bonds. Recognition by guardians requires closer connections to the lived experiences of their peers. It is essential to exchange knowledge and experiences with other guardians, to have support of family and friends, to access knowledge produced by research as well as policies that ensure the rights and inclusion of their children.^5^

Social networks are formed by the interweaving of interpersonal bonds, where synergistic interactions take place. Primary social networks involve interpersonal relationships consisting of family, kinship, friendship, neighborhood and work ties, which provide support or containment. Secondary networks, on the other hand, can be informal or formal and include third-sector or non-profit institutions and market-based entities.^6^

This study focuses on the primary social network, due to the complexity and depth of existing ties and relationships. Families of transgender people often face rejection, disapproval and social isolation, along with psychological and physical burdens, in addition to a lack of informational support regarding social and health rights, health care and advocacy.^7^

This study aimed to analyze the primary social network of mothers, fathers or guardians of transgender children or adolescents, motivated by the following guiding questions: What are the characteristics of the structure of the primary social network of mothers, fathers or guardians of transgender children and adolescents? What are its functions and dynamics?

## METHODS

This was a qualitative, descriptive and exploratory study based on the analysis of social network dynamics.^6^ This report follows the guidelines of the consolidated criteria for reporting qualitative research.^8^

This study was conducted at a national level and supported by the National LGBT Alliance, the non -governmental organization (NGO) Mothers for Diversity and the National Association of Transvestites and Transsexuals (*Associação Nacional de Travestis e Transexuais* – ANTRA); the Transgender Care and Support Space at the Hospital das Clínicas /Pernambuco state; and the Comprehensive Care Center for Black and LGBT Population/Jaboatão dos Guararapes/Pernambuco state, and T Outpatient Clinic for Transgender People in Porto Alegre, Rio Grande do Sul, state. These services have interdisciplinary teams working in management, healthcare and social movement actions. These teams provide health education, conduct research, produce materials and offer support to transgender people and their families. 

Mothers, fathers or guardians of transgender children and adolescents living in the states of Ceará, Pernambuco, São Paulo, Espírito Santo, Minas Gerais, Paraná, Rio de Janeiro, Rio Grande do Sul, and the Federal District took part in this study.

People up to 9 years of age were considered children. Those between 10 and 19 years of age were considered adolescents.^9^

Data collection took place between August and October 2021. Participants were selected using the snowball sampling technique, aimed at reaching a hard-to-access population due to the significant social stigma and cultural, social and historical barriers that disregard the existence of transgender people and outline transphobia. ^10,11^

Initial contact was made with professionals or representatives from organizations that supported the study; they made recommendations and shared the interview scheduling forms with potential respondents. Individuals identified comprised the zero wave and, given the difficulty in accessing this target population, referred other participants to comprise the subsequent waves. There were four participants withdrew after three contact attempts.

The characterization tool and interview guide were validated by experts, researchers, and professionals with experience with transgender people and their families. A pre-test was also conducted, via self-completion, with eight people. Two of these participants had their pre-tests included after agreeing to participate in the video interview via Google Meet, and recorded by the researchers.

Participants received the informed consent form and the characterization form via WhatsApp, which they completed on the previously agreed day and time. After signing the consent form, interviews were conducted via Google Meet by trained researchers. The interviews lasted approximately one hour.

Social network exploration involved the construction of maps for didactic visualization of the network components and established ties, called Rousseau’s map.^6^

We decided to build the map after the interviews, based on participants’ verbalized responses, who provided the necessary information . The participants received a summary of the responses and the map of their social network including descriptions and legends, in order to validate the information collected.

Individual interviews were conducted. Audio and/or video were recorded and the interviews were fully transcribed, and pseudonyms (flower species) were used to protect the participants identities. The empirical material was organized according into three dimensions: structure, function and dynamics, with an emphasis on the most prominent network components.

The construction of the social network structure of the study participants based on network components, ties and indicators was carried out in five steps: (i) synthesis of information; ( ii ) construction of Rosseau’s maps; (iii) validation by participants; (iv) identification of indicators; and (v) construction of a consolidated map.

The Rosseau’s map was designed to enable the visualization of the social network structures, through the analysis of the function and dynamics of primary and secondary networks, considering ties and bonds established.

The analysis of function and dynamics required a complementary in-depth analysis of the text corpus using the maps and statements, which were subjected to thematic content analysis. A floating reading of the corpus was then performed, which was organized according to the responses of all participants.^12-14^

The corpus was analyzed using the *Interface de R pour les Analyses Multidimensionelles de Textes et de Questionnaires* (IRaMuTeQ) software version 0.7, which provided hierarchical descending classification, displayed in a dendrogram.^14^ Lexical analysis was also performed to complement the content analysis.^15^ The classes obtained from the analyses were interpreted to define the thematic axes**.** The thematic axes were condensed into thematic categories and the inductive analysis was performed, following the social network dynamics method.^6^

The project was submitted to and approved by the Research Ethics Committee of the Escola de Enfermagem de Ribeirão Preto da Universidade de São Paulo, Opinion No. 4,567,837, on March 2, 2021, 30405720.4.0000.5393.

## RESULTS

Thirty mothers, two fathers and one grandmother of trans children and adolescents, with an average age of 46 years, took part in the study. Thirteen had a postgraduate degree; eleven had completed higher education; six had incomplete higher education; two had completed high school; and one had incomplete technical education.

**Figure 1:**
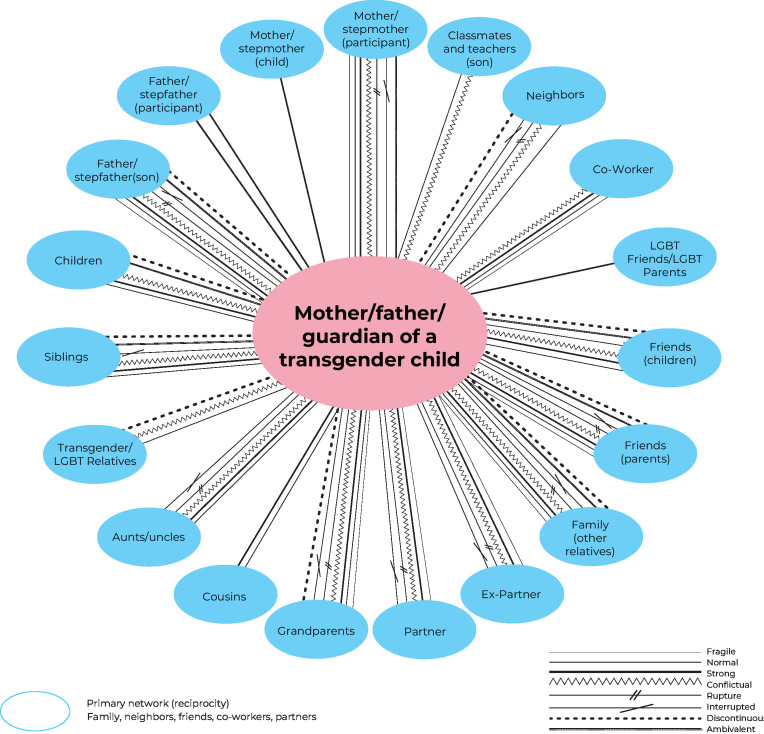
Structure of primary social networks and bonds established with guardians and their transgender descendants

In total, 33 individual maps, comprised of primary networks, were developed and analyzed (Figure 1).

**Figure 2:**
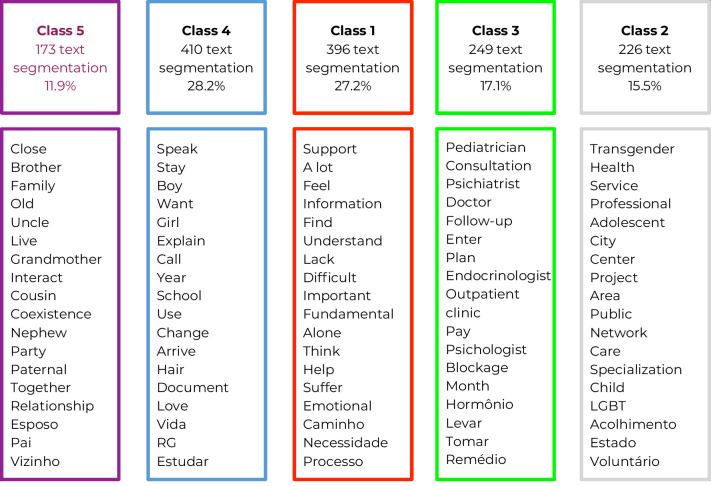
Most significant words in each class analyzed in the classification analysis descending hierarchy

The text corpus was divided into 1,681 text segments, comprising 4,553 words that occurred 58,416 times. The descending hierarchical classification retained 86.5% of the total text segments, aggregated into five classes (Figure 2). These classes were grouped into two thematic categories following thematic analysis: “Family as the center of the network and the challenges to achieving transgender autonomy” (Classes 5, 4 and 1) and “Strengths and weaknesses of secondary networks for comprehensive health care” (Classes 2 and 3).

It could be seen that the guardian-child/adolescent transgender social configuration forms the first network of responsibility and affection for the transgender person. This network is not composed by choice but serves a function of dependency for care and protection. The bonds established in this binomial showed shared strengths and weaknesses in the challenges related to the recognition of gender identity, with the nuclear family being the center of the social network, which will be presented below.

It was found that normality relationships between some members of the primary network were evident among those who reacted naturally in the family contexts in which transgenderism had been disclosed.

The statements also reveal that being a transgender person became a reference for other relatives in similar situation to disclose their own gender identity and/or sexual orientation, becoming a source of pride (Box 1).

**Box 1:** Excerpt from participants’ speeches

**Focus on the bonds of normality** *[...] my mother was 66 years old and had a great understanding . My brother was more questioning and raised the issue to his psychologist. She opened many doors within the family. After (my daughter’s) transition, my nieces identified as LGBT,* which caused a revolution within the family. (Calendula) *[...] this journey always involves me, my wife and my daughter, usually I take the lead, because my schedule is more flexible [...] we research, exchange ideas among ourselves and decide what is best for everyone [...] due to [this] demand, I changed jobs [...] we have not transitioned yet, we hope that prejudiced people will move away.* (Saint George’s Sword) *The closest people are my father, my sister and me [...] there are children in the condominium and he made friends, these children know that he is a trans boy and accept him as he is. He made friends,* having already transitioned *[...]* (Sunflower) *[...] People respect him a lot, and my niece even gave him the first piece of cake at her party and even said it was for her favorite cousin. It was a minute of silence. He has some friends, but no contact*. (Amaryllis)
**Focus on strong bonds** *he was very well accepted, included, and respected in all settings, largely because I assert this; people look to the mother’s stance, and she gives feedback according to how I position myself*. (Carolina) *When my daughter transitioned, these were relationships that needed time [...] today, these are relationships that have restored what they were before, as if there had been no interruption. These are strong and healthy relationships.* (Pansy) *[...] they are good relationships. They are my friends from school, college, work, they are different groups, but they are all good partners and my whole family.* (Mayflower) *I looked for the LGBT group there in (city where I live) and then I explained my story [...] They said : look, talk to (trans man), he’s a really cool person, he’ll be able to help you [...] and we became friends.* (Iris)
**Focus on weak bonds** *My mother doesn’t accept a weak relationship like that, because she treats him like a girl and since he doesn’t want to argue with her, he accepts it. Not anymore, but I used to argue a lot [...] (With my father) it’s a very weak relationship, because they don’t spend much time together.* (Hydrangea) *[...] my family accepted it very well, but, in his father’s family, things probably won’t be like that, so it’s more a matter of prejudice, because, with knowledge, I think prejudice decreases*. (Peristery) *[...] you are such in dark place in those moments, yes, marriages can fall apart, children can leave . You are so lost, there is no comfort for you, how are you going to support your husband and your other daughter who is crying?* Someone needs to figure it out fast, and for me, it happened quicker. (Ros) *[...] the most difficult process was with my boyfriend, I already realized it before my son told me, I already realized some things and I tried to adapt, but he resisted a lot, so it took longer with him, but when we talked, the support was immediate.* (Heliconia) *[...] my husband’s family is more traditional [...] they say that it’s been difficult for them, that they’re still working on this idea.* ( Lily)
**Focus on conflicted bonds** *The father (conflictual relationship) [...] mistreats my daughter because of someone who was important in her life (trans friend). He comes to my gate and calls (birth name). Then I let him shout outside until he calls (social name).* (Iris) *[...] the father is in the Navy, (daughter) lived with him until she was 9 years old, and what he did was shout when she spoke in a more feminine voice : ‘speak like a man’.* (Orchid) If you’re a prejudiced parent, your child is going to be prejudiced too because you’re teaching them that. The other kids treated him just fine, but one dad specifically questioned him on the street, and he came home really upset, crying [...] (Mandacaru) *[...] I distanced myself from those who were weak, I had to cut them off, why would I want a relationship with a person who was becoming weak at that moment?* (Morning Glory) *[...] my mother, who until recently was already very close, right? But, when she realized that we weren’t doing things the way she wanted, she cut ties.* (Lily) *I had three specific problems with three evangelical people, and one of them was my aunt [...] it was very difficult because I don’t believe that religion will separate us, and she said, ‘you sold your daughter’s soul to the devil’.* (Begonia) *The paternal grandfather [...] he sends money, he sends gifts, but it wasn’ t much of a gift either [...] and now it’s practically zero.* (Iris)

It is worth highlighting the testimony of a father who changed his work routine to provide care for his transgender son. The father actively participated in peer groups, contributed to research, collected literature on the subject and took part in scientific events and policy-making meetings, aimed to acquire information and contribute to support strategies, as well as understand his role, receive support and support his son (Box 1).

Strong bonds were identified primarily in the mother figure’s role in supporting the child, which facilitated connections with other transgender people and strengthened friendship bonds that had initially been interrupted.

Strong bonds were also identified in relationships between a few close friends and family members of transgender children – who responded naturally to the recognition of their descendant’s gender identity.

The social networks were small – comprising up to nine members - with few members of the network interacting with each other to provide support.^6^ Despite being limited, the support proved relevant, even enabling other family members to reveal their own LGBT+ identity. (Box 1).

Fragile bonds​ were described in relationships with family, friends, relatives and colleagues as potential bonds to be interrupted. Distancing, neglect and emotional restraint were more frequently observed, particularly in the figure of the cisgender man (Box 1).

It could be seen that conflictual ties were motivated by situations of transphobia, rather than recognition of gender identity. These also converged towards severed, broken and discontinuous ties. The normalization of recognizing transgender identity was more evident among transgender and cis children in the school and neighborhood context. Children appeared more cohesive, as they did not question transgenderism as adults did. However, other parents expressed transphobic behaviors regarding the trans friends of their cisgender children, with an impact on education and the tendency towards transphobia (Box 1).

The recognition of gender identity was a critical point in alliances and transgressions within the dynamics of primary social networks, being decisive in the construction or breakdown of support and advocacy behaviors. The “social transition” of the transgender child or adolescent culminates in the “family transition” within a mutual context of resistance and struggle.

## DISCUSSION

The analysis of the social network of guardians of transgender children and adolescents adds a complex layer to discussions about policies and care, with a strong presence of the role of parents or guardians in the latter. Legal guarantees, such as the right to a social name and anti-discrimination laws, have enabled progress. Similarly, the expansion of health services, psychological support for transgender people and their families, and the strengthening of social networks at various levels have been fundamental in promoting adequate inclusion and support.^16^

Guardians of transgender children and adolescents represent the center of the social network in providing affection and care, ties that are crucial for building other relationships within the social network. The nuclear family is often exposed to hostility and harassment from relatives^17,18^ and neighbors.^18^

Recognition of gender identity was found to be permeated by denial and doubt. The “social transition” of the transgender person was also permeated by a “family transition” according to the participants in this study, also understood as recognition. For them the term “transition” highlights the stages before and after the recognition and disclosure of gender identity. These stages can be considered critical moments for the consolidation of bonds.

The decision to support their transgender descendants sometimes resulted in the potential loss of friends^3,19-24^ and recurring challenges in maintaining bonds with friends, family, and neighbors.^20^

In this study, the limited participation of fathers may indicate lesser involvement of the father figure in the functions of taking responsibility for the care of descendants. Weaknesses in support were more frequent in male relatives, such as fathers, partners and paternal family members.^25^

The mother figure sometimes needs to recognize the gender identity of the child in advance to strengthen the dialogue with the extended family.^26^ This study and the literature also show that mothers have a greater physical, emotional and organizational burden,^27^ Given that their position is considered a reference point for decisions that impact relationships. In addition, discouragement and conflicts with other generations, such as grandparents,^21,28^ as well as friends or family members, who believed that providing support could contribute to significant harm to the child, were evident.^24^

In this and other studies, the approach of guardians to transgender people resulted in strengthened bonds of friendship, emotional and informational support. Knowledge building was more expressive through peer-to-peer knowledge exchange and less evident in bonds with healthcare professionals, as they did not know how to address transgender-specific needs.^7^

This study highlights the fragility of bonds between the components of the social network due to the lack of recognition and information about transgender people. In this sense, in the health field, knowledge production for health education can be done through: books and stories about gender identity of transgender children and their families; research; guidelines and strategies for transgender people and their families, and it is essential that they are available not only online, but also in physical spaces with public access.^17^ Informational support for guardians has been more evident in relationships with gay or transgender friends.^29-30^

The inclusion of participants linked to civil society support networks may have suppressed the experience of those who do not recognize the gender identity of their descendants, and represents an important limitation of this study. The family perspective may have excluded the context of transgender children and adolescents living in shelters and potential participants who are not linked to NGOs.

The primary social network showed ineffective support with limited support and need for strengthening. This analysis is an important tool for structuring policies, care pathways, and educational technologies aimed at comprehensive care within the context of primary healthcare, with an emphasis on the family and its social network. The purpose is to design individualized projects aimed at transgender health from childhood. 

The networks presented limited support to guardians of transgender children and adolescents and needed to be strengthened. The analysis of bonds between network components is an essential tool for planning care and health actions, structuring policies, and constructing a trans-specific care pathway.
