# Supplementary material for: Social network for guardians of transgender children and adolescents
Source: Epidemiol Serv Saude. 2025 Jan 13;33(spe1):e2024290. doi: 10.1590/S2237-96222024v33e2024290.especial.en (PMC11734605; doi:10.1590/S2237-96222024v33e2024290.especial.en)
Supplement: Translation

# Translation: Rede social das pessoas responsáveis por crianças e adolescentes transgêneros

**DOI:** 10.1590/S2237-96222024v33e2024290.especial.pt

**Authors:** Paula Daniella de Abreu, Ana Beatriz Marques Valença, Gilberto da Cruz Leal, Diogo Henrique Mendes da Silva, Pedro Fredemir Palha, Ednaldo Cavalcante de Araújo, Jaqueline Garcia de Almeida Ballestero, Sandra Aparecida de Almeida, Jordana de Almeida Nogueira, Aline Aparecida Monroe

**Affiliations:** 1 Universidade de São Paulo, Escola de Enfermagem de Ribeirão Preto, SP, Brasil; 2 Universidade Federal de Pernambuco, Departamento de Enfermagem, Recife, PE, Brasil; 3 Universidade Federal da Paraíba, Departamento de Enfermagem em Saúde Coletiva, João Pessoa, PB, Brasil; 4 Universidade Federal da Paraíba, Departamento de Enfermagem Clínica, João Pessoa, PB, Brasil

**Keywords:** Rede Social, Pessoas Transgênero, Identidade de Gênero, Criança, Adolescente

## Abstract

**Objetivo:**

Analisar a rede social de mães, pais ou responsáveis por crianças ou adolescentes transgêneros.

**Métodos:**

Trata-se de estudo qualitativo, fundamentado no referencial de rede social, com enfoque na rede primária. O estudo foi desenvolvido no Brasil por meio de entrevistas *online* entre agosto e outubro de 2021. Participaram 30 mães, dois pais e uma avó de crianças ou adolescentes transgêneros. Realizou-se análise de conteúdo, modalidade temática, com auxílio do software IRaMuTeQ.

**Resultados:**

A temática “A família enquanto centro da rede e os desafios para o alcance da autonomia trans” emergiu a partir das análises. A família configurou-se como primeira rede, com maior responsabilização. Revelaram-se vínculos frágeis e conflituosos com familiares, amigos, colegas e vizinhos, destacando a figura do homem.

**Conclusão:**

As redes apresentaram limitações no apoio e necessidade de fortalecimento, sendo a análise uma importante ferramenta na assistência, estruturação de políticas e linha de cuidado transespecífica.

## Contribuições do estudo

**Principais resultados:**

As redes de apoio dessas famílias, especialmente a família imediata, desempenham papel relevante e apresentam fragilidades, como vínculos conflituosos e falta de suporte adequado de outros membros da comunidade.

**Implicações para os serviços:**

Abordagem mais estruturada e específica no atendimento de famílias de crianças e adolescentes transgêneros é necessária. Os serviços de saúde devem fortalecer o apoio à família, oferecer assistência específica e capacitar profissionais de saúde para o cuidado dessa população.

**Perspectivas:**

Pesquisas futuras devem explorar intervenções que minimizem conflitos e fortaleçam laços, além de avaliar o impacto de políticas transespecíficas na autonomia e bem-estar de crianças e adolescentes transgêneros, orientando a criação de políticas públicas mais inclusivas.

## INTRODUÇÃO

Os desafios enfrentados pelos responsáveis por crianças ou adolescentes transgênero, ou trans, consiste inicialmente no processo de reconhecimento da transgeneridade. Tal desconhecimento pode resultar em experiências de negação e transfobia, potencializadas por sentimentos de dúvidas, impotência diante dos desafios, do medo, da tristeza e da culpabilização.^1-3^ Esses sentimentos também podem estar associados à experienciação de “perda ambígua”, entendida como a perda psicológica e identitária de uma pessoa que ainda está presente fisicamente.^4^

A identidade de gênero trans pode modificar os vínculos familiares existentes. O reconhecimento pelos responsáveis demanda aproximação com a vivência de seus pares. É imprescidível a troca de saberes e experiências com outros responsáveis, o suporte de familiares e amigos, o auxílio do conhecimento produzido por pesquisas e acesso a políticas de acolhimento e garantia dos direitos de seus filhos.^5^

As redes sociais constituem no entrelaço de vínculos interpessoais, nas quais ocorrem interações sinérgicas. As redes sociais primárias implicam as relações interpessoais compostas por laços familiares, parentais, de amizade, vizinhança e trabalho que exercem a função de apoio ou contenção. As redes secundárias, por outro lado, podem ser informais ou formais e incluem instituições de terceiro setor ou sem fins lucrativos e as de mercado.^6^

Este estudo foca a rede social primária, devido à complexidade e ao aprofundamento dos vínculos e das relações existentes. A família da pessoa trans usualmente enfrenta rejeições, desaprovações e isolamento social, sobrecarga psicológica e física, além da falta de apoio informativo sobre os direitos sociais e de saúde, cuidados a saúde e advocacia.^7^

O presente estudo busca analisar a rede social primária das mães, pais ou responsáveis por crianças ou adolescentes transgênero e foi motivado pela seguinte questão norteadora: Como se caracteriza a estrutura da rede social primária das mães, pais ou responsáveis por crianças e adolescentes transgênero? Quais as funções e dinâmicas? 

## MÉTODOS

Trata-se de um estudo qualitativo, descritivo e exploratório fundamentado na análise de dinâmicas de rede social.^6^ O presente relato se baseou nas orientações do *consolidated criteria for reporting qualitative research*.^8^

O estudo foi desenvolvido em âmbito nacional e contou com o apoio da Aliança Nacional LGBT, da organização não governamental (ONG) Mães pela Diversidade e da Associação Nacional de Travestis e Transexuais (Antra); do Espaço de Cuidado e Acolhimento Trans do Hospital das Clínicas/Pernambuco; e, do Núcleo de Atenção Integral à População Negra e LGBT/Jaboatão dos Guararapes/Pernambuco e do Ambulatório T para pessoas Trans de Porto Alegre. Esses serviços possuem equipes interdisciplinares que atuam no campo da gestão, da assistência à saúde e das ações dos movimentos sociais. Tais equipes instruem quanto à educação em saúde, produzem pesquisas e materiais e acolhem as pessoas transgêneros e os familiares destas. 

Participaram do estudo mães, pais ou responsáveis por crianças e adolescentes transgêneros residentes no Ceará, Pernambuco, São Paulo, Distrito Federal, Espírito Santo, Minas Gerais, Paraná, Rio de Janeiro e Rio Grande do Sul.

Pessoas com até 9 anos de idade foram consideradas crianças. Aquelas que possuíam entre 10 e 19 anos foram consideradas adolescentes.^9^

A produção dos dados empíricos ocorreu entre agosto e outubro de 2021. A seleção dos participantes se deu através da técnica bola de neve (*snowball*). O intuito era de reunir a população de interesse de difícil acesso devido ao marcante estigma social e às barreiras culturais, sociais e históricas que desconsideram a existência das pessoas transgêneros e delineiam a transfobia.^10,11^

Iniciou-se o contato com os profissionais ou representantes das organizações que deram apoio ao estudo; estes fizeram indicações e compartilharam os formulários de agendamento das entrevistas a possíveis respondentes. As pessoas indicadas compuseram a onda zero e, dada a dificuldade de acesso a esse público-alvo, indicaram outros participantes para compor as ondas subsequentes. Houve quatro desistências de participação, sendo consideradas após três tentativas de contato.

O instrumento de caracterização e o roteiro da entrevista foram validados por especialistas, pesquisadores e profissionais com experiência com o público transgênero e familiares. Houve também um pré-teste, via autopreenchimento, com oito pessoas. Duas destas tiveram seus pré-testes incluídos após aceitarem participar da entrevista por vídeo via Google Meet, e registradas pelos pesquisadores. 

Os participantes receberam via WhatsApp o termo de consentimento Livre e esclarecido e o formulário de caracterização para preenchimento no dia e no horário previamente pactuados. Após assinatura do termo, foram conduzidas entrevistas no Google Meet. Estas foram conduzidas por pesquisadores previamente treinados. As entrevistas duraram, em média, uma hora.

A exploração de redes sociais se deu pela construção de mapas para visualização didática dos componentes da rede e vínculos estabelecidos, denominado mapa de Rousseau.^6^

Optou-se pela construção do mapa após as entrevistas. Isso foi feito com base nas respostas verbalizadas pelos participantes que conduziram as informações necessárias. Os participantes receberam uma síntese das respostas e o mapa da sua rede social com a descrição e a legenda, a fim de validar as informações coletadas.

Entrevistas individuais foram realizadas. Áudio e/ou vídeo foram gravados e as entrevistas foram transcritas na íntegra, sendo utilizados codinomes (espécies de flores) para resguardar a identidade dos participantes. O material empírico foi organizado segundo as três dimensões: estrutura, função e dinâmica, com ênfase nos componentes da rede mais emergentes. 

A construção da estrutura da rede social dos participantes do estudo em função de componentes da rede, laços e indicadores foi realizada em cinco etapas: (i) síntese das informações; (ii) construção dos mapas de Rosseau; (iii) validação pelos participantes; (iv) identificação dos indicadores; e (v) construção do mapa consolidado.

O mapa de Rousseau foi construído para possibilitar a visualização da estrutura das redes sociais, por meio da análise da função e dinâmica das redes primárias e secundárias, considerando laços e vínculos estabelecidos.

A análise da função e dinâmica demandou, de forma complementar, análise aprofundada do córpus textual por meio dos mapas e depoimentos que foram submetidos à análise de conteúdo, modalidade temática. Foi realizada, então, leitura flutuante do córpus, que foi organizado pelas respostas de todos os participantes.^12-14^

O córpus foi analisado com auxílio do *software* Interface de R pour les Analyses Multidimensionelles de Testes et de Questionnaires (IRaMuTeQ) versão 0.7, onde foi realizada a classificação hierárquica descendente, disponibilizada em dendograma.^14^ Obteve-se a análise léxica para complementar à análise de conteúdo.^15^ As classes obtidas a partir das análises foram interpretadas para definição dos eixos temáticos**.** Os eixos temáticos foram condensados em categorias temáticas e procedeu-se à análise indutiva, seguindo método de dinâmica de rede social.^6^

O projeto foi submetido e aprovado pelo Comitê de Ética em Pesquisa da Escola de Enfermagem de Ribeirão Preto da Universidade de São Paulo, por meio Parecer nº 4.567.837, de 2 de março de 2021, 30405720.4.0000.5393. 

## RESULTADOS

Trinta mães, dois pais e uma avó de crianças e adolescentes trans, com idade média de 46 anos, participaram do estudo. Treze possuíam pós-graduação; onze, ensino superior completo; seis, ensino superior incompleto; dois, ensino médio completo; e um, ensino técnico incompleto.

Ao todo, 33 mapas individuais, compostos pelas redes primárias foram elaborados e analisados (Figura 1).

**Figura 1:** Estrutura das redes sociais primárias e dos laços estabelecidos com os responsáveis e seus descendentes trans

O córpus textual foi dividido em 1.681 segmentos de texto, relacionando-se 4.553 palavras que ocorreram 58.416 vezes. A classificação hierárquica descendente reteve 86,5% do total de segmentos de texto, agregados em cinco classes (Figura 2). Tais classes foram agrupadas em duas categorias temáticas após análise temática:” “A família enquanto centro da rede e os desafios para o alcance da autonomia trans” (Classes 5, 4 e 1) e “Fortalezas e fragilidades das redes secundárias para atenção integral em saúde” (Classes 2 e 3). 

**Figura 2:** Palavras mais significativas em cada classe analisada na análise de classificação hierárquica descendente

Observou-se que a conformação social responsável-criança/adolescente trans consiste na primeira rede de responsabilização e afetividade da pessoa trans em que não há escolha de composição, mas há função de dependência para os cuidados e a proteção. Os laços constituídos nesse binômio revelaram fortalezas e fragilidades compartilhadas nos desafios para o reconhecimento da identidade de gênero, sendo a família nuclear o centro da rede social, aos quais serão apresentados a seguir.

Constatou-se que os vínculos de normalidade entre alguns membros da rede primária foram evidenciados entre aqueles que reagiram com naturalidade nos contextos familiares em que a transgeneridade havia sido divulgada. 

As falas também revelam que ser trans se tornou referência para que outros parentes na mesma condição optassem por divulgar a própria identidade de gênero e/ou orientação sexual, tornando-se motivo de orgulho (Quadro 1).

**Quadro 1:** Recorte do discurso dos participantes

**Foco nos vínculos de normalidade** *[...] minha mãe tinha 66 anos e teve um superentendimento. Meu irmão foi mais questionador, levou a questão para o psicólogo dele. Ela abriu muitas portas dentro da família, as minhas sobrinhas depois da transição de (filha) se declararam LGBTs, fez uma revolução na família.* (Calêndula) *[...] essa busca é sempre eu, minha esposa e minha filha, geralmente eu que tomo a frente, porque os meus horários são mais flexíveis [...] a gente pesquisa, troca entre nós e decide pelo que é melhor para todo mundo [...] devido [a] essa demanda, mudei de serviço [...] não transicionamos ainda, espera-se um afastamento das pessoas preconceituosas.* (Espada de São Jorge) *As pessoas mais próximas são o meu pai, eu e a irmã [...] no condomínio tem crianças e ele fez amizade, essas crianças sabem que ele é um menino trans e aceitam ele dessa forma. Ele fez amizade, ele já tinha feito a transição [...]* (Girassol) *[...] As pessoas respeitam bastante ele, e a minha sobrinha que até deu o primeiro pedaço do bolo para ele na festa dela e ainda falou que era para o primo que ela mais gosta. Foi um minuto de silêncio. Tem alguns amigos, mas não tem contato*. (Amarílis)
**Foco nos vínculos forte** *[...] ele foi muito bem aceito , incluído, respeitado em todos os âmbitos, até porque eu imponho muito isso, as pessoas esperam muito como a mãe vai se impor, ela vai me dar o* feedback *de acordo com o meu posicionamento.* (Carolina) *Na fase que a minha filha transicionou, foram relações que precisaram de um tempo [...] hoje são relações que restauraram o que era antes, como se não tivesse tido essa interrupção. São relações fortes e saudáveis.* (Amor-perfeito) *[...] são boas relações. São meus amigos que vêm de escola, faculdade, trabalho, são grupos diferentes, mas todos são bem parceiros e minha família toda.* (Flor-de-maio) *Eu procurei o coletivo LGBT lá em ( município que reside) e aí eu expliquei a minha história [...] Falaram assim: olha, conversa com (homem trans), ele é uma pessoa bem legal, vai poder te ajudar [...] e a gente foi ficando amigo.* (Íris)
**Foco nos vínculos fracos** *A minha mãe não aceita, (uma relação) fraca assim, porque ela trata como menina e como ele não quer brigar com ela , ele aceita. Agora mais não, mas eu já discuti bastante [...] (Com o pai) é uma relação bem fraca, porque eles não têm muito convívio.* (Hortênsia) *[...] minha família aceitou muito bem, mas, na família do pai dele, as coisas provavelmente não vão ser assim, então mais é a questão do preconceito, porque, a partir do conhecimento, eu acho que diminui o preconceito*. (Peristéria) *[...] você está tão nas trevas que nessas horas, sim, casamentos podem ser desfeitos, filhos podem ir embora. Você está tão perdido, não tem conforto para você, como que você vai acolher o teu marido e a outra filha que chora? Alguém tem que achar o entendimento mais rápido, o entendimento chegou mais rápido para mim.* (Rosa) *[...] o processo mais difícil foi meu namorado , eu já percebia antes de ser dito pelo meu filho , eu já percebi algumas coisas e eu já tentava me adaptar, mas ele resistia muito, aí foi mais demorado com ele, mas na hora que eu tive a conversa o apoio foi imediato.* (Helicôndia) *[...] a família do meu marido que são mais tradicionais [...] eles falam que para eles está sendo difícil, que eles ainda estão trabalhando essa ideia.* (Flor-de-lis)
**Foco nos vínculos conflituosos** *O pai ( relação conflituosa) [...] maltrata a minha filha por causa de uma pessoa que foi fundamental na vida dela (amigo trans). Ele chega aqui no meu portão e chama (nome de registro). Aí eu deixo ele gritando lá fora até ele chamar a (nome social).* (Íris) *[...] o pai é um militar de Marinha, (filha) conviveu com ele dentro de casa só até os 9 anos, e o que ele fazia era gritar quando ela falava com a voz mais afeminada: ‘fala como homem’.* (Orquídea) *Se você é um pai, uma mãe preconceituosa, seu filho vai ser preconceituoso, porque você está incutindo isso na personalidade dele. E as crianças tratavam ele igual, mas um pai especificamente questionou ele na rua, ele chegou muito triste em casa, chorou [...]* (Mandacaru) *[...] as que eram frágeis eu me distanciei, eu tive que cortar, para que que eu ia querer uma relação com uma pessoa que naquele momento estava fragilizando?* (Jitirana) *[...] a minha mãe que até um pouco tempo atrás já estava bem próxima, né? Mas, quando percebeu que a gente não estava fazendo como ela gostaria, ela cortou os laços.* (Lírio) *Eu tive três problemas específicos que foram três pessoas evangélicas , e aí uma delas é a minha tia [...] foi muito difícil porque eu não acredito que a religião vai separar a gente, e ela falou assim ‘você vendeu a alma da sua filha pro diabo’.* (Begônia) *O avô paterno [...] ele manda dinheiro, ele manda o presente, mas já não era muito presente também [...] e agora sim tá praticamente zero.* (Íris)

Cabe destacar o depoimento de um pai que preferiu optar pela mudança na rotina de trabalho para ofertar cuidados ao filho trans. O pai participou ativamente no grupo de pares e contribuiu com pesquisas, levantou literatura sobre o tema e participou de eventos científicos e encontros para elaboração de políticas com a finalidade de adquirir informações e contribuir com estratégias de acolhimento, bem como para compreender sua função, ter apoio e apoiar o filho (Quadro 1). 

Identificou-se que os vínculos de natureza forte apresentaram maior destaque no posicionamento da figura materna diante o apoio ao filho(a), o que refletiu na aproximação com outras pessoas trans e no fortalecimento de vínculos de amizades que, em um primeiro momento, foi interrompido. 

Os vínculos fortes foram identificados nas relações entre restritos amigos e familiares dos filhos trans – que reagiram com naturalidade ao reconhecimento da identidade de gênero do descendente. 

As redes sociais possuíam amplitude pequena - até nove integrantes - e poucos membros da rede interagiam entre si para relações de apoio.^6^ Apesar de restrito, o apoio mostrou relevante inclusive por viabilizar que outros familiares revelassem ser LGBT+ (Quadro 1).

Os vínculos considerados frágeis foram descritos nas relações entre familiares, amigos, parentes e colegas como prováveis vínculos a serem interrompidos. Foram evidenciados o distanciamento, as negligências e a contenção afetiva, de forma mais frequente, na figura do homem cisgênero (Quadro 1).

Observou-se que vínculos conflituosos foram motivados por situações de transfobia, e não de reconhecimento da identidade de gênero. Também convergiram para laços interrompidos, rompidos e descontínuos. A naturalização no reconhecimento da identidade de gênero trans foi mais evidente entre as crianças trans e cis no contexto escolar e de vizinhança. As crianças se apresentaram mais coesas, pois não questionavam a transgeneridade como faziam pessoas adultas. Outros pais, por sua vez, expressaram comportamentos transfóbicos em relação às amizades trans dos seus filhos cisgêneros com impacto na educação e na tendência à transfobia (Quadro 1).

O reconhecimento da identidade de gênero foi ponto crítico, nas alianças e nas transgressões nas dinâmicas das redes sociais primárias, sendo definidora na construção ou no rompimento das condutas de acolhimento e advocacia. A “transição social” da criança ou do adolescente trans culmina com a “transição familiar” no contexto mútuo de resistência e luta.

## DISCUSSÃO

A análise da rede social dos responsáveis por crianças e adolescentes transgêneros adiciona camada complexa às discussões sobre políticas e cuidados, nesse último caso com forte presença do papel dos pais ou cuidadores. Garantias legais, como o direito ao nome social e leis antidiscriminação, possibilitaram progressos. Da mesma forma, a ampliação dos serviços de saúde, o apoio psicológico para pessoas trans e seus familiares, e o fortalecimento das redes sociais em diversos níveis têm sido fundamentais para promover inclusão e suporte adequados.^16^

Os responsáveis por crianças e adolescentes transgêneros representam o centro da rede social no provimento de afeto e cuidados laços determinantes para construção de outras relações com a rede social, sendo o núcleo familiar por vezes exposto a hostilidade e assédio de parentes^17,18^ e vizinhos.^18^

O reconhecimento da identidade de gênero se mostrou permeado por negação e dúvida. A “transição social” da pessoa trans também foi permeada por uma “transição familiar” segundo os participantes deste estudo, também compreendido como reconhecimento. O termo “transição” para estes demarca as etapas antes e após o reconhecimento e a revelação da identidade de gênero. Essas etapas podem ser consideradas momentos críticos para a consolidação de vínculos.

A decisão de apoiar seus descendentes trans, por vezes, resultou na potencial perda de amigos^3,19-24^ e em recorrentes desafios nos vínculos com amigos, família e vizinhos.^20^

Neste estudo, a restrita participação de pais pode ser um indicativo de menor atuação da figura paterna nas funções de responsabilização no cuidado dos descendentes. As fragilidades de apoio foram mais frequentes em familiares do gênero masculino, como pais, parceiros e família paterna.^25^

A figura materna, por vezes, necessita reconhecer antecipadamente a identidade de gênero do(a) filho(a) para fortalecer o diálogo com a família extensa.^26^ Este estudo e a literatura também apresenta que as mães possuem maior sobrecarga física, emocional e organizacional,^27^ visto que seu posicionamento é tido como referência para decisões que impactam as relações. Além disso, foi evidente o desencorajamento e conflitos com outras gerações, os avós,^21,28^ além de amigos ou familiares, que acreditavam que apoiar poderia contribuir com danos significativos ao filho(a).^24^

Neste e em outros estudos a aproximação dos responsáveis com pessoas trans resultou em fortalecimento de vínculos de amizade, apoio emocional e informativo. A construção do conhecimento foi mais expressiva na troca de saberes entre pares e menos evidente no vínculo com profissionais da saúde, pois estes não sabiam acolher as demandas trans.^7^

Este estudo evidencia a fragilidade de vínculos entre os componentes da rede social devido ao falta de reconhecimento e informações sobre pessoas transgêneros. Neste sentido, no âmbito da saúde a produção do conhecimento para educação em saúde pode se dar por meio de: livros e histórias sobre identidade de gênero das crianças trans e suas famílias; pesquisas; diretrizes e estratégias para a pessoa trans e família, sendo imprescindível estar disponível não apenas online, mas também em espaços físicos de acesso público.^17^ O apoio informativo aos responsáveis têm sido mais evidente nas relações com amigos *gays* ou trans.^29-30^

A inclusão dos participantes vinculados a redes de apoio da sociedade civil organizada pode ter suprimido a experiência daqueles que não reconhecem a identidade de gênero dos seus descendentes, e representa importante limitação ao presente estudo. A perspectiva familiar pode ter suprimido o contexto de crianças e adolescentes trans que vivem em abrigos e possíveis participantes que não estão vinculados a ONG.

A rede social primária apresentou apoio ineficaz com limitado apoio e necessidade de fortalecimento. A análise é uma importante ferramenta para estruturação de políticas, linha de cuidado e tecnologias educacionais com vistas ao cuidado integral no contexto da atenção primária à saúde com ênfase na família e sua rede social. A finalidade é traçar projetos singulares voltados à saúde trans desde a infância.

As redes apresentaram limitado apoio aos responsáveis por crianças e adolescentes transgêneros e necessidade de fortalecimento. A análise dos vínculos entre os componentes da rede é uma importante ferramenta no planejamento do cuidado e ações em saúde, na estruturação de políticas e construção de uma linha de cuidado transespecífica.
